# Here and There

**Published:** 1898-02

**Authors:** 


### HERE AND THERE.

Chicago's Latest Effort to Raise Money for Use in Improving its Streets and Parks.—Chicago people are interested in the new wheel-tax lately levied by the municipal authorities of that city. The owner of a one-horse carriage must take out a license, paying one dollar a year; a two-horse outfit will cost two dollars a year, and so on. A bicycle-tax ordinance is also agitating the town. Many people claim that these ordinances are unconstitutional. Suburban riders say that the new law may cause the arrest of many non-residents. The new vehicle-label has been selected by City Clerk Loeffler. The tag, which is to be attached to the harness of the horse, is made of high-grade German silver, the background oxidized, and all raised surfaces polished bright. For one-horse vehicles the figure 1 will appear, raised in the centre in silver on a red enamel ground; for two-horse, the figure 2 will appear on a blue ground; for the three-horse, the figure 3 on a yellow ground; for the four-horse vehicle, the figure 4 on a green ground. The label is attached by metal clamps, which will be fastened to the harness.

This tax is to be used exclusively in the construction and maintenance of good roads.

An investigation on Texas fever, which may result in much profit to cattle-owners and breeders in this State, is being conducted at the Veterinary Department of the University of Pennsylvania. Texas fever is a disease which attacks cattle going from Northern States to Southern States, and causes death. This practically prevents the sending of fine-grade cattle from this State to Texas, where there is a good market for them.

The Fleshers' Trade Protection Committee of Glasgow, Scotland, has sent a circular to shipowners saying that after December 1st the members of their organization will not purchase United States or Canadian live-stock carried by shipping companies who transport live-stock for importers not belonging to the organization.

A white colt, by Palentine, with red ears, has been dropped at Oak Hill Farm, near Nashville, and hundreds have visited the equine freak.

Queen Victoria owns a dress manufactured entirely of spiders' webs.

Good horses, wanted by buyers either for the track or road, are said to be worth 20 per cent. more than they were last year at this time.

A two-year-old colt was gelded April 20, 1895, near Toledo, Ohio. Two days later he was turned out with a mare. April 1st last the mare dropped a horse foal.

The cutting of a rope, to which a horse was tethered, by a railroad train, at Niagara Falls, was followed by the animal dashing into the waters and being swept over the Horseshoe Falls to destruction.

Henry Ward Beecher once said that society owes to the horse a debt of gratitude a thousand times greater than it does to thousands of men who abuse him. He has ministered to progress; has made social intercourse possible when otherwise it would have been slow and occasional, or altogether impossible; he has virtually extended the strength of man, augmented his speed, doubled his time, decreased his burdens, and, becoming his slave, has relieved him from drudgery and made him free. For love's sake, and for the sake of social life, for eminent moral reasons, the horse deserves to be bred, trained, and cared for with scrupulous care. The teaching of men how to do it has been left too long to men who look upon the horse as an instrument of gambling gains or of mere physical pleasure.

The late Sir Arthur Helps said: "Whenever I see horses suffering from too tight a check-rein I know that the owner is unobservant, cruel, or pompous. He is unobservant, or he would see that his horses are suffering. He is ignorant, or would know that a horse loses much of his power of pulling and cannot recover himself if he stumbles; and he is cruel if, observing and knowing, he does not remedy it. He is pompous and vulgar if he prefers that his horses rear their heads on high and rattle their trappings to being dealt with humanely and reasonably. When I look at the coat-of-arms on these carriages I know who are the greatest fools in the upper classes. The idiot and brute of a coachman likes to sit behind these poor, tortured, faithful martyrs, with their reined-up heads, but his master ought to know better."

Many Philadelphia veterinarians will remember the trick-horse "Mazeppa," owned and exhibited by Mr. Hugh Maguire, who has just obtained a verdict of $13,454 in the United States Circuit Court from the New England Railroad Company, Mazeppa having been killed in a railroad accident.

"Never tell me that a horse has not a soul," says the Chicago *Herald*. "That soul may not bear a through tag to eternity, but while it abides it holds the principles that pertain to immortals. The other day I was passing along the street with a great American beauty in my hand, the stem of which was nearly as long as an alpen-stock. As I strolled by a horse standing on the corner of State and Monroe the rose that I carried brushed the animal's nose, and he turned lovingly after it."

A horse supposed to be suffering from rabies, following the appearance of a rabid dog at Smyrna, Delaware, attacked several other horses, and when approached by its owner, turned, and the owner only escaped in his flight by the animal being seized with a paroxysm and falling to the ground. The animal was shot before further damage was done.

Many veterinarians received a full year's subscription to the JOURNAL for 1898, or a Glass book on *Diseases of the Dog*, in their stockings, as a reminder of Santa Claus of former years.

Dr. A. W. Bitting, of Purdue University, discussed municipal control of dairy-herds, and showed its necessity, at the Indiana Dairymen's Convention in December. Only nine cities in Indiana have milk-inspection, and the system is not uniform or practical.

Dr. J. N. Hurty, Secretary of the State Board of Health, referring to the exclusion of 12,000 pounds of milk daily from the Chicago market, because of too dirty dairies and the presence of typhoid fever, and the loss that thus fell on Lake County farmers, urges the forming of local sanitary associations to govern the production, care, and proper sanitation of dairies, and thus to back up the State Board in their efforts to secure proper inspection of dairies, and, by controlling the dangers of unwholesome milk, increase the sale of pure milk, and thus aid the dairyman.

A Philadelphia angora cat, losing by death her four kittens, pre-empted two puppies from a neighboring kennel, and is now proving a true foster-mother to the puppies.

The Grand Rapids Veterinary College is associated with the Medical College of the same name as its Veterinary Department. Some eleven students are in attendance, others are expected to enter. The term is two years of six months each. Dr. H. Rutherford, formerly of the Detroit Veterinary College, is director.

Flemington, N. J., reports an outbreak of rabies among a flock of some fifty sheep, following the advent of a rabid dog among them.

Three-year-old steers are selling in Palisade, Nev., at $30 per head.

The Dominion of Canada Cattle-breeders' Association in December resolved to ask for such modification of the quarantine law as will admit cattle from Great Britain on the certificate of veterinary inspectors in that country that they have passed the tuberculin-test.

Dr. Carl Schlatter, of Zurich, Switzerland, has successfully accomplished the triumph of major surgery in removing the stomach in its entirety from a woman, with recovery and an improved condition of the patient over her previous physical health for many months. What a revelation!

Germany announces paper bottles.

Chief Government reindeer herdsman William A. K. Jeilman, now in Alten, Norway, has been cabled to send six hundred reindeer for draught purposes in the Klondike region.

Russia has half the world's horses.

The use of condensed meats in the Alaskan gold-regions is strongly urged by the Government. Their easy transportation and keeping qualities strongly recommend them.

Paris has an official rat-catcher.

The citizens of Cedar Rapids, Iowa, will have the opportunity of purchasing milk from one of the most complete sanitary dairies of our country. Thoroughly inspected and tested cattle, scrupulously clean animals, stables, and attendants, and the most improved appliances for handling, Pasteurizing, and delivery of milk, insure a pure and healthful supply.

Nearly $5,000,000 worth of patent medicines are exported from the United Kingdom each year.

The Chicago horse-show was a grand financial success. The receipts are said to have exceeded the disbursements by some twenty-two thousand dollars. The total premiums reached the sum of forty-five thousand dollars. It will prove a great boon to equine interests all over the country. The selection of veterinary inspectors this year was an innovation, and will now, we are assured, become a recognized feature.

Mr. George McLaughlin has an interesting article in the November 25th issue of the *Independent*, descriptive of the work and future of the veterinarian.

